# Supplementary material for: Reconciling Mining with the Conservation of Cave Biodiversity: A Quantitative Baseline to Help Establish Conservation Priorities
Source: PLoS One. 2016 Dec 20;11(12):e0168348. doi: 10.1371/journal.pone.0168348 (PMC5173368; doi:10.1371/journal.pone.0168348)
Supplement: S1 Dataset — (ZIP) [file pone.0168348.s002.zip › Taxa/Serra Norte/SN_2013/N5S/Lista N5S-24.pdf]

CAVIDADE N5S-0024

Classe	Ordem	Fam/Outros	Gên/Outros	Espécie	S-U	U-S	Zon.
Arachnida	Araneae	Araneidae		jovem	X	X	E
Arachnida	Araneae	Filistatidae		jovem		X	E
Arachnida	Araneae	Pholcidae	*Mesabolivar*	*eberhard*	X	X	E
Arachnida	Araneae	Scytodidae	*Scytodes*	jovem	1	1	E
Arachnida	Araneae	Theridiosomatidae	*Plato*	sp.4	X	X	E
Chilopoda				jovem		X	E
Entognatha	Collembola	Paronellidae		sp.1	X		E
Insecta	Coleoptera	Chrysomelidae		sp.19		X	E
Insecta	Diptera	Cecidomyiidae	Cecidomyiinae	sp.	X	X	E
Insecta	Diptera	Tipulidae	Tipulinae	sp.	X	X	E
Insecta	Heteroptera	Reduviidae	Emesinae	jovem		1	E
Insecta	Hymenoptera	Formicidae	*Wasmannia*	sp.1	X		E
Insecta	Neuroptera	Mantispidae	*Plega*	sp.		2	E
Insecta	Orthoptera	Phalangopsidae	*Paraclodes*	sp.	1		E
Insecta	Psocoptera	Psyllipsocidae	*Psyllipsocus*	sp.2		X	E
Gastropoda	Pulmonata	Systrophiidae	*Happia*	sp.	X		E
Mammalia	Chiroptera	Emballonuridae	*Peropteryx*	sp.		1	E
